# Glioma identification from microRNA biomarkers using machine learning

**DOI:** 10.3389/fsysb.2026.1771910

**Published:** 2026-06-09

**Authors:** Rakesh Kanth Andugala, Alyson Cieslik, Maria Braoudaki, Iosif Mporas

**Affiliations:** 1 School of Physics, Engineering and Computer Science, University of Hertfordshire, Hatfield, United Kingdom; 2 School of Health, Medicine and Life Sciences, University of Hertfordshire, Hatfield, United Kingdom

**Keywords:** brain cancer, glioma, machine learning, meningioma, MicroRNA biomarkers

## Abstract

Gliomas are the most aggressive malignant brain tumours, occurring mostly in adults and accounting for approximately 80% of central nervous system malignant tumours. Traditional diagnostic methods are both invasive and expensive, thus accurate, minimally invasive, and cost-effective early detection is vital to guide personalised treatment plans. MicroRNAs (miRNAs) are stable non-coding RNAs detectable in various body fluids (e.g., serum, plasma, and cerebrospinal Fluid (CSF)) that regulate gene expression and influence cellular processes; their dysregulation is a significant factor in cancer development. This makes them a promising biomarker for glioma classification. In this article, we present a glioma identification methodology from miRNA data using machine learning (ML) followed by data analysis for miRNA biomarker investigation. A machine learning pipeline is applied to classify glioma from controls as well as from meningioma samples using miRNA expression data obtained by four Gene Expression Omnibus (GEO) datasets (GSE112264, GSE113486, GSE113740, GSE139031). After preprocessing, five feature selection techniques (LASSO, mRMR, ReliefF, RFE, and RF importance) were employed. Six machine learning algorithms (LR, KNN, DT, RF, SVM, XGB) were used for classification with and without SMOTE oversampling. Performance was assessed after 5-fold cross-validation, in terms of accuracy, F1-score, precision, recall, and area under the curve (AUC). The results showed that in binary classification (glioma vs controls) all models achieving up to 100% accuracy, and in multi-class classification (glioma vs meningioma vs controls) up to 100% F1-score was achieved with both KNN and XGB classifiers. The top-ranked miRNAs were also analysed and compared with biomarkers previously known from the literature. Seven miRNAs were identified as potential biomarkers, namely the miR-125a-3p, miR-4276, miR-4648, miR-4763-3p, miR-663a, miR-6784-5p and miR-873-3p, and were independently validated on the GSE211692 dataset.

## 1 Introduction

Gliomas are a heterogeneous group of primary brain tumours arising from glial cells and are classified by the World Health Organization (WHO) according to their histopathological and molecular characteristics. (Louis et al., 2021). These tumours present symptoms, including headaches, seizures, and cognitive deficits, driven by their infiltrative growth (IJzerman-Korevaar et al., 2018). Despite aggressive treatments, such as surgical resection, radiotherapy, and chemotherapy, patient prognosis remains poor, underscoring the need for early and accurate diagnosis. Conventional diagnostic methods, including magnetic resonance imaging (MRI) and histopathological biopsy, face limitations (Li K. et al., 2024). MRI lacks specificity to differentiate gliomas from other brain lesions, while biopsy, although definitive, is invasive, costly and carries procedural risks that can often delay therapeutic decision-making. Recent studies highlight microRNAs (miRNAs), small non-coding RNAs that regulate gene expression, as promising minimally invasive biomarkers for glioma diagnostics (Zhou et al., 2018). MiRNAs exhibit tumour-specific expression profiles in biofluids (Anelli et al., 2021), such as blood (serum or plasma), saliva, urine, and cerebrospinal fluid, offering high sensitivity and specificity in distinguishing gliomas from other brain lesions or other neurological conditions. However, developing reliable computational models using miRNA expression data is challenging due to their high dimensionality, the typically small sample sizes in available training datasets, class imbalances, and variability in data collection and processing (Muhamed Ali et al., 2018). To address these issues, this article presents a machine learning pipeline to identify miRNA biomarkers for glioma classification, using online miRNA expression datasets from Gene Expression Omnibus (GEO) (Edgar et al., 2002). Our pipeline employs feature selection and class balancing with Synthetic Minority Over-sampling Technique (SMOTE) to identify miRNA biomarkers by employing both binary (glioma vs controls) and multi-class (glioma vs meningioma vs controls) classification, using several machine learning (ML) algorithms. The miRNAs found to be top ranked in glioma identification were then analysed and compared with other biomarkers known from the literature.

## 2 Related works

### 2.1 miRNAs in glioma classification

Previous research on glioma identification from molecular data and previous studies on cancer classification using miRNAs. Specifically, regarding glioma classification using omics data, (Si et al., 2022), glioma was distinguished from Primary Central Nervous System Lymphoma (PCNSL) by analysing serum miRNA expression profiles from blood serum using Least Absolute Shrinkage and Selection Operator (LASSO) and Support Vector Machine - Recursive Feature Elimination (SVM-RFE), identifying ten diagnostic miRNAs. In (Li et al., 2023), glioma was distinguished from controls based on the within-sample relative expression orderings of miRNAs, reporting 100% accuracy. In (Slimene et al., 2022), glioma was classified among four other cancer types (Breast invasive carcinoma (BRCA), Stomach and Esophageal carcinoma (STES), Pan-kidney cohort (KIPAN), and Ovarian Serous Cystadenocarcinoma (OV)) by analysing miRNA expression profiles (Reads Per Million (RPM) normalised) from tissue samples, using DeepInsight (Sharma et al., 2019) for feature extraction and ResNet50 (Koonce and Koonce, 2021) for classification, achieving 92.9% prediction accuracy. In (Ni et al., 2024), low-grade glioma (LGG) was classified from five other cancer types (including lung adenocarcinoma (LUAD), kidney pan-cancer (KIPAN), thyroid carcinoma (THCA), sarcoma (SARC), and esophageal carcinoma (ESCA)), based on miRNA expression profiles from human tissue samples, MiRS-HF deep learning framework with two graph convolutional networks (LAGCN and GCNCC) and feature weighting, achieving accuracy of 95.6% for KIPAN, 94.8% for ESCA, 77.9% for SARC, 72.8% for THCA, 63.8% for LGG and 63.2% for LUAD and identifying 20 miRNA biomarkers. In (Kaczmarek et al., 2021), LGG was classified from 12 other cancer types (Bladder urothelial (BLCA); Breast invasive (BRCA); Cervical/endocervical (CESC); Head and neck squamous cell (HNSC); KIPAN; LGG; Liver hepatocellular (LIHC); LUAD; Lung squamous cell (LUSC); Prostate (PRAD); STES; THCA), by analysing patient-matched miRNA and mRNA expression data from The Cancer Genome Atlas (TCGA) (Tomczak et al., 2015) tissue samples, using the Graph Transformer Network (GTN) to model miRNA-mRNA interactions, achieving 93.56% classification accuracy. In (Wang T. et al., 2021), LGG (Grade2,3) by analysing miRNA, mRNA, and DNA methylation data from tissue samples, using the proposed Multi-Omics Graph convolutional NETworks (MOGONET) deep learning framework combining graph convolutional networks and View Correlation Discovery Network (VCDN), achieving ROSMAP dataset (of Alzheimer’s disease (AD) patients vs. normal control (NC)) (accuracy (ACC): 0.815, F1 score (F1): 0.821, area under the receiver operating characteristic curve (AUC): 0.874), LGG (ACC: 0.816, F1: 0.814, AUC: 0.840), BRCA (ACC: 0.829, F1 weighted: 0.825, F1 macro: 0.774), and KIPAN (matched adaptive group-regularized ridge regression (GRridge) (Van De Wiel et al., 2016) in AUC, surpassed in ACC and F1. In (Waspada et al., 2017), miRNA expression data (1881 features) from 22 cancer tissue types and normal samples were classified using supervised machine learning methods, decision tree, naïve Bayes, neural network (Artificial Neural Network), and deep learning (Deep Neural Network). Deep learning achieved 91.49% accuracy in the multi-class scenario. Binary classifications (breast or cervical cancer vs. normal) yielded up to 100% accuracy with deep learning and neural networks. In (Pyman et al., 2018), glioma was distinguished from 26 other cancer types and non-neoplastic samples, using miRNA expression profiles from human tissues, employing stacked autoencoders and a multi-layer perceptron, achieving 94.73% for multi class classification accuracy. In (Laplante and Akhloufi, 2020), glioma was classified from 20 anatomical sites (grouped from 27 cancer location) using miRNA stem-loop expression data from TCGA (Tomczak et al., 2015) human tissue samples, employing a deep neural network and an overall F1 score of 96.88%, with 18 of 20 classes having an F1 score greater than 90%. In (Noviandy et al., 2023), LGG and glioblastoma (GB were classified by analysing molecular components, genetic mutations, and clinical features from TCGA (Tomczak et al., 2015) human tissue samples, using ensemble machine learning with Genetic Algorithm Logistic Regression (GA-LR) (Hosseini, 2020), Boruta (Kursa and Rudnicki, 2010), and CatBoost Feature Importance (CFI) (Dhananjay and Sivaraman, 2021) feature selection, achieving 86% F1-score. In (Lokeswaran et al., 2024), classification of five brain tumour types (medulloblastoma, glioma, rhabdoid tumour, primitive neuro-ectodermal tumour (PNET), and normal) was presented, using gene expression data from human tissue samples, with Principal Component Analysis (PCA) and Pearson correlation feature selection, achieving 90% multiclass classification accuracy with LR. In (Oberoi et al., 2023), LGG, GB, and their subtypes (oligodendroglioma, astrocytoma, glioblastoma, and mixed glioma) were classified by analysing gene mutation and clinical data from human tissue samples, achieving 90.70% accuracy for grade classification using an ensemble of Logistic Regression (LR), Support Vector Machine (SVM), and K-Nearest Neighbors (KNN) with Mutual Information feature selection and 84.88% accuracy for subtype classification using a Bagging Classifier with Mutual Information feature selection. In (Wang et al., 2023), classification of LGG into grade 2 and grade 3 was performed by analysing multimodal data (miRNA, mRNA, and DNA methylation) from human tissue samples, utilising a proposed deep learning framework, Dual Trustworthy Mechanism for Classification (DTMC), reporting 89.8% AUC.

### 2.2 miRNAs in other cancer types classification

Beyond brain cancer, miRNAs have also been utilized to distinguish other malignancies. For instance, in (Kaczmarek et al., 2022) neoplastic from non-neoplastic breast and skin tissue samples were classified using miRNA expression profiles, while AUC of the Deep Cancer Classifier (DCC) (Pyman et al., 2018) was 99.9% for breast cancer and 97.1% for skin cancer. In (Pal and Rami, 2024), classification between normal and breast cancer was performed on miRNA expression patterns from human blood samples, achieving up to 100% accuracy with Artificial Neural Networks (ANN) and Minimum Redundancy Maximum Relevance (mRMR). In (Slimene et al., 2021) breast invasive carcinoma was distinguished from control using miRNA RPM expression data from human tissue samples, with SVM achieving 0.99 accuracy, 1.0 sensitivity, and 0.99 specificity. In (Mehejabin et al., 2020) breast cancer samples were classified from normal ones using miRNA expression data from human tissue samples, with SVM achieving 99.23% accuracy. In (Li et al., 2022) a prognostic risk model classifying cervical cancer patients into high or low risk categories was constructed based on miRNA expression and clinical data from TCGA (Tomczak et al., 2015) human tissue samples, using Random Forest (RF) for feature selection and multivariate Cox regression, achieving 0.94 AUC on 5-year survival prediction. In (Kantamneni et al., 2024) subtypes of lung cancer (adenocarcinoma, squamous cell carcinom a, and Small Cell Lung Cancer (SCLC)) along with healthy controls were classified based on miRNA expression profiles from GEO (Edgar et al., 2002), using a late fusion of FNN and DNN (Feedforward Neural Network and Deep Neural Network) model, achieving 90.7% accuracy. In (Andreini et al., 2022) breast cancer classification from healthy controls was performed based on miRNA expression data from human tissue samples, using a two-stage SVM and Random Forest (RF) machine learning approach, achieving 0.9901 accuracy.

## 3 Methodology

**FIGURE 1:**
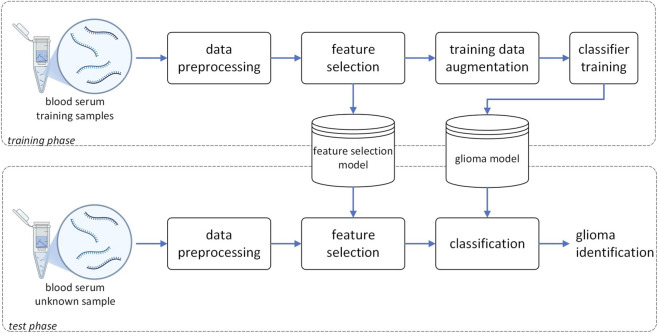
Block diagram of the methodology for glioma identification from miRNA data. SMOTE is applied only to the training folds within cross-validation; the test fold remains unchanged.

The methodology followed for glioma identification is presented in this section, including a description of the datasets used and the processing steps of the employed architecture, which is illustrated in Figure 1.

### 3.1 Datasets and preprocessing

The miRNA expression data were acquired from four GEO (Edgar et al., 2002) datasets: GSE112264 (Urabe et al., 2019), GSE113486 (Usuba et al., 2019), GSE113740 (Yamamoto et al., 2020), and GSE139031 (Ohno et al., 2019). All cohorts were derived from human serum samples and processed using the same microarray platform (Toray 3D-Gene Human miRNA V21, GPL21263), which ensured consistency across the integrated data. Therefore, explicit batch-effect correction methods (e.g., ComBat (Johnson et al., 2007) or limma removeBatchEffect (Ritchie et al., 2015)) were not applied to avoid potential over-correction, which could inadvertently remove biologically meaningful variation.

**FIGURE 2:**
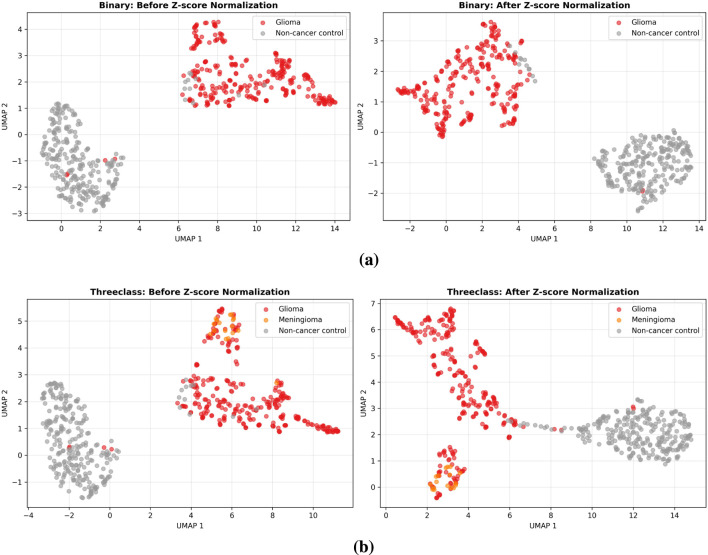
UMAP visualization before and after z-score normalization. **(a)** Before z-score normalization. **(b)** After z-score normalization.

To further mitigate potential inter-study variability and batch effects, we evaluated the impact of different preprocessing strategies, specifically comparing Min-Max scaling and z-score standardization. We implemented z-score standardization as it provided better stability across datasets. Figure 2 illustrates the UMAP visualization before and after z-score normalisation.

This processed dataset supported two experimental configurations: a binary classification setup involving 552 samples (285 glioma and 267 non-cancer controls), and a multi-class setup (glioma vs. meningioma vs. non-cancer controls) comprising 569 samples (285 glioma, 17 meningioma, and 267 non-cancer controls). Each sample consists of 2,565 miRNA values, with the distribution across datasets and classes detailed in Table 1.

**TABLE 1:** Data distribution in the evaluation and validation datasets.

Dataset	Glioma	Meningioma	Non-cancer controls	Total samples
GSE112264	50	-	-	50
GSE113486	40	-	100	140
GSE113740	25	-	10	35
GSE139031	170	17	157	344
Total (evaluation data)	**285**	**17**	**267**	**569**
GSE211692 (validation data)	241	-	5,643	5,884

An independent external validation cohort, GSE211692 (Matsuzaki et al., 2023), was additionally obtained from GEO. This dataset contains human serum miRNA expression profiles generated on the GPL21263 platform. After label mapping and alignment with the trained feature space, the external cohort included 5,884 samples in total, 241 glioma samples and 5,643 non-cancer control samples. The GSE211692 dataset was used only for independent validation testing.

### 3.2 Feature selection

Five feature ranking algorithms were employed to perform dimensionality reduction. These are (i) the Least Absolute Shrinkage and Selection Operator (LASSO) (Tibshirani, 1996) based on L1 regularisation, (ii) the Minimum Redundancy Maximum Relevance (mRMR) (Peng et al., 2005), (iii) the ReliefF (Kononenko, 1994), (iv) the Recursive Feature Elimination (RFE) (Guyon et al., 2002) and (v) the Random Forest (RF) (Breiman, 2001). To prevent information leakage and overfitting, feature selection was performed independently within each iteration of the cross-validation process. Features were selected solely based on the training folds, ensuring that the test fold remained completely unseen during the dimensionality reduction phase.

### 3.3 Machine learning algorithms

Six machine learning algorithms (ML) were used to train glioma identification models. These are the Logistic Regression (LR) (Hastie et al., 2009), the K-Nearest Neighbours (KNN) (Cover and Hart, 1967), the Decision Tree (DT) (Quinlan, 1986), the Random Forest (RF) (Breiman, 2001), the Support Vector Machine (SVM) (Cortes and Vapnik, 1995) with linear kernel, and the Extreme Gradient Boosting (XGB) (Chen et al., 2015). Each model was trained on various miRNA feature subsets obtained after the feature selection process (tested dimensionalities: 5, 10, 20, 50, 100, 250, 500, 750, 850, 1,000, 1,100, 1,500, 2,000, 2,565), and hyperparameters were optimized using grid search following a 5-fold stratified cross-validation protocol.

### 3.4 Class imbalance handling

Class imbalances were addressed using the Synthetic Minority Oversampling Technique (SMOTE) (Chawla et al., 2002). SMOTE was used to generate synthetic samples for the minority class only for the training subsets, while the test subsets remained unchanged with the original samples only.

However, it is important to note that while SMOTE addresses numerical imbalance, it generates synthetic samples by interpolating between neighbouring minority-class samples in feature space. For very small classes such as meningioma (n=17) , this does not introduce new biological variation and may smooth or blur genuine within-class heterogeneity (e.g., potential substructure or outliers), which can affect biological interpretability. Consequently, this scarcity remains a primary limitation that may impact the model’s ability to generalize complex biological features compared to more prevalent classes.

### 3.5 Performance evaluation

Five metrics of performance were estimated across all evaluated glioma identification models, namely, the classification Accuracy, the F1-score, the Precision, the Recall, and the Area Under the Receiver Operating Characteristic Curve (AUC).

## 4 Results

The methodology for glioma identification presented in Section 3 was evaluated using two experimental setups, binary classification (glioma vs. controls) and multi-class classification (glioma vs. meningioma vs. controls), with and without SMOTE oversampling.

### 4.1 Binary classification

The binary classification (glioma vs. controls) results without SMOTE data augmentation are tabulated in Table 2. The best performing feature selection (FS) algorithm and the corresponding number of selected miRNA features are shown in the second column and ‘*’ stands for all five feature ranking algorithms.

**TABLE 2:** Binary Classification (glioma vs controls) Results without SMOTE.

ML method	Best FS	Class	ACC	F1-score	Precision	Recall	AUC
LR	* (10)	Glioma	100.00	100.00	100.00	100.00	100.00
​	​	Controls	100.00	100.00	100.00	100.00	100.00
​	​	All	100.00	100.00	100.00	100.00	100.00
KNN	* (10)∖mRMR	Glioma	100.00	100.00	100.00	100.00	100.00
​	​	Controls	100.00	100.00	100.00	100.00	100.00
​	​	All	100.00	100.00	100.00	100.00	100.00
DT	* (10)	Glioma	99.91	99.82	99.65	100.00	99.91
​	​	Controls	99.82	99.81	100.00	99.63	99.88
​	​	All	99.82	99.82	99.81	99.83	99.91
RF	* (10)∖mRMR	Glioma	100.00	100.00	100.00	100.00	100.00
​	​	Controls	100.00	100.00	100.00	100.00	100.00
​	​	All	100.00	100.00	100.00	100.00	100.00
SVM	* (10)	Glioma	100.00	100.00	100.00	100.00	100.00
​	​	Controls	100.00	100.00	100.00	100.00	100.00
​	​	All	100.00	100.00	100.00	100.00	100.00
XGB	* (10)	Glioma	100.00	100.00	100.00	100.00	100.00
​	​	Controls	100.00	100.00	100.00	100.00	100.00
​	​	All	100.00	100.00	100.00	100.00	100.00

As can be seen in Table 2, all evaluated ML algorithms achieved their best performance when using the top 10 ranked miRNA features from LASSO, ReliefF, mRMR, RFE or RF importance. Among them, hsa-miR-663a was commonly selected across all five feature selection methods. Several others, such as hsa-miR-125a-3p (ReliefF, mRMR, RFE, RF importance), hsa-miR-6784-5p (LASSO, mRMR, RFE, RF importance), and hsa-miR-873-3p (LASSO, ReliefF, mRMR, RFE), were shared among the 4 FS methods, while the remaining miRNAs differed across the different evaluated algorithms. Compared with the discussion section (top 100 features), hsa-miR-663a and hsa-miR-6784-5p were identified in both the top 10 and top 100 feature analyses. Except DT, all other evaluated ML algorithms classified correctly all miRNA samples.

The binary classification results using SMOTE data augmentation are tabulated in Table 3. As can be seen in Table 3, applying SMOTE did not change the classification performance, as the results remain identical to those obtained without SMOTE. This was expected since in the binary classification setup there are 285 glioma samples and 267 control samples, i.e., the dataset is already relatively balanced.

**TABLE 3:** Binary Classification (glioma vs controls) Results with SMOTE.

ML method	Best FS	Class	ACC	F1-score	Precision	Recall	AUC
LR	* (10)	Glioma	100.00	100.00	100.00	100.00	100.00
​	​	Controls	100.00	100.00	100.00	100.00	100.00
​	​	All	100.00	100.00	100.00	100.00	100.00
KNN	* (10)∖mRMR	Glioma	100.00	100.00	100.00	100.00	100.00
​	​	Controls	100.00	100.00	100.00	100.00	100.00
​	​	All	100.00	100.00	100.00	100.00	100.00
DT	* (10)	Glioma	99.82	99.82	99.65	100.00	99.90
​	​	Controls	99.82	99.81	100.00	99.63	99.91
​	​	All	99.82	99.82	99.81	99.83	99.91
RF	* (10)∖mRMR	Glioma	100.00	100.00	100.00	100.00	100.00
​	​	Controls	100.00	100.00	100.00	100.00	100.00
​	​	All	100.00	100.00	100.00	100.00	100.00
SVM	* (10)	Glioma	100.00	100.00	100.00	100.00	100.00
​	​	Controls	100.00	100.00	100.00	100.00	100.00
​	​	All	100.00	100.00	100.00	100.00	100.00
XGB	* (10)	Glioma	100.00	100.00	100.00	100.00	100.00
​	​	Controls	100.00	100.00	100.00	100.00	100.00
​	​	All	100.00	100.00	100.00	100.00	100.00

### 4.2 Multi-class classification

The multi-class classification (glioma vs. meningioma vs. controls) results without SMOTE data augmentation are tabulated in Table 4. As can be seen in Table 4, the classification results without SMOTE demonstrate consistently high performance across all models, with KNN and XGB classifying all samples correctly. However, the limited meningioma sample size (n=17) should be also considered in the interpretation of the results due to the potential risk of overfitting. KNN performed best using the 100 top ranked miRNAs as ranked by RF importance, while XGB performed best using the 500 top ranked miRNAs as ranked by LASSO. When meningioma samples are included in the multi-class setup, to maintain high classification accuracy 100 miRNA features are required compared to the 10 features in the case of binary classification setup.

**TABLE 4:** Multi-class classification (glioma vs meningioma vs controls) results without SMOTE.

ML method	Best FS	Class	ACC	F1-score	Precision	Recall	AUC
LR	ReliefF (1,000)	Glioma	99.88	99.82	100.00	99.65	100.00
​	​	Meningioma	99.82	97.14	94.44	100.00	99.99
​	​	Controls	100.00	100.00	100.00	100.00	100.00
​	​	All	99.82	98.99	98.15	99.88	99.99
KNN	RF (100)	Glioma	100.00	100.00	100.00	100.00	100.00
​	​	Meningioma	100.00	100.00	100.00	100.00	100.00
​	​	Controls	100.00	100.00	100.00	100.00	100.00
​	​	All	100.00	100.00	100.00	100.00	100.00
DT	RF (1,000)	Glioma	98.77	98.77	98.94	98.60	99.96
​	​	Meningioma	98.95	83.33	78.95	88.24	99.74
​	​	Controls	99.82	99.81	100.00	99.63	100.00
​	​	All	98.77	93.97	92.63	95.49	99.90
RF	RFE (850)	Glioma	99.47	99.48	98.96	100.00	99.99
​	​	Meningioma	99.47	90.32	100.00	82.35	99.93
​	​	Controls	100.00	100.00	100.00	100.00	100.00
​	​	All	99.47	96.60	99.65	94.12	99.97
SVM	ReliefF (850)	Glioma	99.65	99.65	99.65	99.65	99.99
​	​	Meningioma	99.65	94.12	94.12	94.12	99.98
​	​	Controls	100.00	100.00	100.00	100.00	100.00
​	​	All	99.65	97.92	97.92	97.92	99.99
XGB	LASSO (500)	Glioma	100.00	100.00	100.00	100.00	100.00
​	​	Meningioma	100.00	100.00	100.00	100.00	100.00
​	​	Controls	100.00	100.00	100.00	100.00	100.00
​	​	All	100.00	100.00	100.00	100.00	100.00

The multiclass classification results using SMOTE data augmentation are tabulated in Table 5. As can be seen in Table 5, applying SMOTE improves classification performance across four out of six models. Compared to Table 4 (without SMOTE), where KNN achieved perfect accuracy with 100 miRNAs and XGB required 500 miRNAs, the use of SMOTE enables both KNN and XGB to reach perfect performance using 100 miRNAs. The top 100 miRNAs selected for KNN using RF feature selection were the same in both with and without SMOTE setups. Moreover, DT, RF, and SVM, which previously required 850–1,000 miRNAs, achieved absolute performance with only 100 features when using SMOTE. This demonstrates the positive effect of SMOTE in improving class balance in ML model training, resulting in reducing the number of required miRNAs to maximise classification performance. Crucially, while SMOTE balances the data numerically, it does not introduce new biological variation; thus, the scarcity of original samples remains a constraint for broader generalization.

**TABLE 5:** Performance of machine learning models for multi-class classification (glioma vs meningioma vs controls) with SMOTE.

ML method	Best FS	Class	ACC	F1-score	Precision	Recall	AUC
LR	RFE (1,000)	Glioma	99.65	99.65	100.00	99.30	99.99
​	​	Meningioma	99.65	94.44	89.47	100.00	99.95
​	​	Controls	100.00	100.00	100.00	100.00	100.00
​	​	All	99.65	98.03	96.49	99.77	99.98
KNN	RF (100)	Glioma	100.00	100.00	100.00	100.00	100.00
​	​	Meningioma	100.00	100.00	100.00	100.00	100.00
​	​	Controls	100.00	100.00	100.00	100.00	100.00
​	​	All	100.00	100.00	100.00	100.00	100.00
DT	RFE (100)	Glioma	99.12	99.12	99.65	98.60	99.97
​	​	Meningioma	99.30	89.47	80.95	100.00	99.78
​	​	Controls	99.82	99.81	100.00	99.63	100.00
​	​	All	99.12	96.13	93.53	99.41	99.91
RF	RF (200)	Glioma	99.30	99.30	99.65	98.95	99.99
​	​	Meningioma	99.93	88.89	84.21	94.12	99.99
​	​	Controls	100.00	100.00	100.00	100.00	100.00
​	​	All	99.30	96.06	94.62	97.69	99.96
SVM	LASSO (100)	Glioma	98.95	98.94	100.00	97.89	99.99
​	​	Meningioma	98.95	85.00	73.91	100.00	99.88
​	​	Controls	100.00	100.00	100.00	100.00	100.00
​	​	All	98.95	94.65	91.30	99.30	99.96
XGB	RF (100)	Glioma	100.00	100.00	100.00	100.00	100.00
​	​	Meningioma	100.00	100.00	100.00	100.00	100.00
​	​	Controls	100.00	100.00	100.00	100.00	100.00
​	​	All	100.00	100.00	100.00	100.00	100.00

For clinical translation, we also considered the trade-off between predictive performance and assay practicality. In our experiments, a panel size of 20 miRNAs provided a good balance between performance and feasibility for qPCR/ddPCR-based workflows, achieving an F1-Score of 0.98, while reducing the panel to 5 miRNAs led to a notable performance drop (F1-Score of 0.88).

**FIGURE 3:**
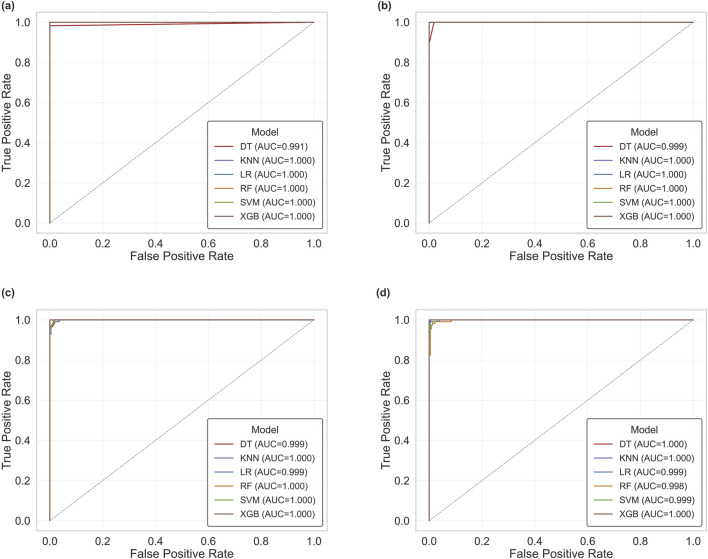
ROC curves of the best performing ML + FS for **(a)** glioma vs controls without SMOTE, **(b)** glioma vs controls with SMOTE, **(c)** glioma vs meningioma vs controls without SMOTE, **(d)** glioma vs meningioma vs controls with SMOTE.

In addition to the AUC scores reported in Tables 2–5, the corresponding Receiver Operating Characteristic (ROC) curves for the best performing ML + FS in binary and multiclass classification, without and with SMOTE, are shown in Figure 3.

## 5 Discussion

**FIGURE 4:**
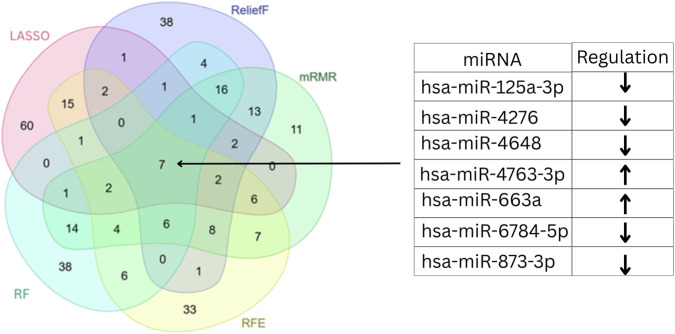
Venn diagram of the number of common miRNAs within the top 100 of each feature ranking algorithm.

Further to the classification analysis for glioma identification presented in Section 4, we conducted additional analysis over the five different feature ranking algorithms to investigate miRNAs that could serve as potential biomarkers for glioma. In specific, the top 100 miRNA features of the binary setup (glioma vs. controls) from each of the LASSO, ReliefF, MRMR, RFE, and RF feature ranking algorithms were compared to identify common ones among them. The Venn diagram showing the number of common miRNAs is shown in Figure 4. Seven common miRNAs were found, namely, the miR-125a-3p, miR-4276, miR-4648, miR-4763-3p, miR-663a, miR-6784-5p and miR-873-3p.

**FIGURE 5:**
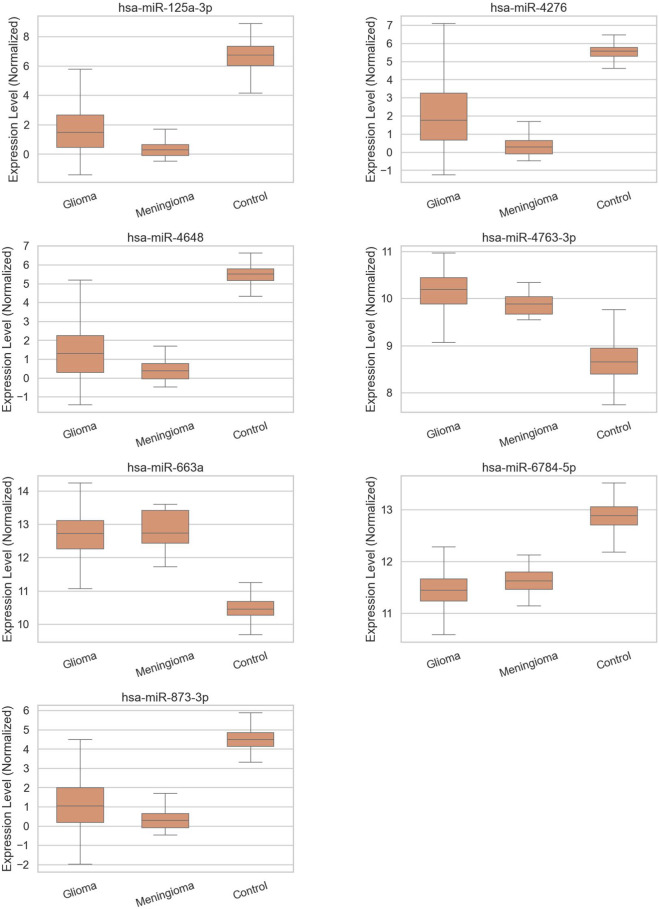
Box plots of the expression level (normalized) of the seven selected miRNA signatures in glioma, meningioma and control samples.

Figure 5 shows the expression levels of the seven selected miRNAs in controls, glioma, and meningioma. The box plots indicate that miR-125a-3p, miR-4276, miR-4648, miR-6784-5p, and miR-873-3p are downregulated in both glioma and meningioma compared with controls, suggesting potential tumour suppressor roles, whereas miR-663a and miR-4763-3p are upregulated in both tumour types, indicating possible oncogenic properties.

To assess the robustness and generalizability of the 7 miRNAs, validation on the independent dataset GSE211692 was performed. The four datasets (GSE112264, GSE113486, GSE113740 and GSE139031) were used for training, without SMOTE (Table 6) and with SMOTE augmentation (Table 7). For reference purposes, except the best ML model using only the 7 miRNA signatures, in Tables 6, 7 we also report the performance of best ML + FS from Section 4 for each ML algorithm on the validation dataset. Despite the substantial class imbalance in the external dataset, the selected miRNA signatures maintained consistently high discriminative ability across classifiers, with AUC values remaining very high and the 7-miRNA model achieving high performance in both settings (LR: 99.68% accuracy without SMOTE in Table 6 and 99.68% with SMOTE in Table 7). Although a slight reduction in some metrics, particularly glioma recall and F1-score, was observed during the independent validation when compared with the evaluation results, the results indicate the robustness and the generalizability of the 7 miRNA signatures when tested on independent data. It is worth noting that the selected miRNA signatures are aligned to or competing in performance the other data-driven ML + FS models while using 3 less miRNAs, and without the requirement for feature selection training.

**TABLE 6:** Top enriched pathways/processes for miR-4276.

Pathway/Process	Merged p -value	Merged FDR
Regulation of transcription, DNA-templated	$2.49\times 10^{-9}$	$6.47\times 10^{-8}$
Protein glycosylation	$1.66\times 10^{-6}$	$1.44\times 10^{-5}$
Cell adhesion	$1.18\times 10^{-6}$	$1.44\times 10^{-5}$
Protein phosphorylation	$2.91\times 10^{-6}$	$1.57\times 10^{-5}$
Phosphorylation	$4.19\times 10^{-6}$	$1.57\times 10^{-5}$
Sphingolipid biosynthetic process	$3.17\times 10^{-6}$	$1.57\times 10^{-5}$
Dendrite morphogenesis	$4.22\times 10^{-6}$	$1.57\times 10^{-5}$
Cell cycle	$9.54\times 10^{-6}$	$2.94\times 10^{-5}$
Nervous system development	$1.17\times 10^{-5}$	$2.94\times 10^{-5}$
Protein transport	$1.10\times 10^{-5}$	$2.94\times 10^{-5}$
Wnt signaling pathway	$1.25\times 10^{-5}$	$2.94\times 10^{-5}$
Circadian rhythm	$1.55\times 10^{-5}$	$3.24\times 10^{-5}$
Rhythmic process	$1.62\times 10^{-5}$	$3.24\times 10^{-5}$
Positive regulation of protein kinase B signaling	$1.89\times 10^{-5}$	$3.51\times 10^{-5}$
Viral process	$2.28\times 10^{-5}$	$3.95\times 10^{-5}$
Golgi organization	$2.55\times 10^{-5}$	$4.15\times 10^{-5}$
Homophilic cell adhesion via plasma membrane adhesion molecules	$3.23\times 10^{-5}$	$4.95\times 10^{-5}$
Axon guidance	$4.21\times 10^{-5}$	$5.76\times 10^{-5}$
Regulation of neurotransmitter secretion	$4.15\times 10^{-5}$	$5.76\times 10^{-5}$
Lipid translocation	$5.64\times 10^{-5}$	$7.33\times 10^{-5}$

**TABLE 7:** Differential expression consistency of candidate novel miRNA biomarkers across GEO datasets (glioma vs. controls), reported as logFC.

Dataset	miR-663a	miR-6784-5p	miR-4276	miR-4648
GSE112264	2.17	−1.145	−3.495	−3.707
GSE113740	2.4997	−1.655	−3.03132	−3.74342
GSE139031	2.198	−1.429988	−3.588789	−3.918159
GSE113486	2.39	−1.37	−2.71	−3.36
GSE211692 (validation data)	2.2501	−1.1435	−3.2127	−3.9943

Further to the seven miRNAs identified as potential biomarkers, linkage with both these 7 miRNAs and additional circulating miRNAs previously reported as glioma-related diagnostic or prognostic biomarkers was performed, to place our findings within the broader biomarker context. In specific, miR-328 was previously identified as part of a panel of miRNAs able to diagnose GB with 91.7% accuracy (Ebrahimkhani et al., 2018). In addition, low expression levels were shown to be associated with poor prognosis in GB patients (Wu et al., 2012). It has also been identified as a diagnostic biomarker of non-small cell lung cancer (Ulivi et al., 2013). Using a bioinformatics approach, miR-4763-3p was identified as a biomarker of diffuse glioma when part of an index composed of three miRNAs (Ohno et al., 2019). miR-873 has previously been shown to be able to serve as a diagnostic biomarker for glioma, with low expression levels linked to poor prognosis (Li et al., 2020). Serum levels of miR-769-3p were able to differentiate glioma from controls with an AUC of 0.925 (Wang et al., 2020). In (Yerukala Sathipati et al., 2022) it was also identified it as part of a signature of 14 diagnostic miRNA biomarkers for bladder urothelial carcinoma, further validating its utility as a cancer biomarker. As previously mentioned, (Li et al., 2023) created a model that utilised the REOs of five pairs of miRNAs to diagnose glioma from non-cancer. Three of the miRNAs included in these pairs, miR-125a-3p, miR-1914-5p and miR-1225-3p, were also identified in this study, supporting the potential utility of these miRNAs as diagnostic biomarkers for glioma. In addition, miR-125a-3p has been identified as a biomarker in pancreatic, colon, prostate and gastric cancer (Salehi et al., 2022; Wang et al., 2017; Damodaran et al., 2021; Li et al., 2025), and miR-1914-5p was previously identified as a diagnostic marker of ovarian cancer using a machine learning approach (Hamidi et al., 2023). Our study also revealed that miR-663a could serve as a potential biomarker for glioma, which, to the best of our knowledge, has not been previously reported. miR-663a has been shown to act as a tumour suppressor in GB. It has been found to be downregulated in GB tissue with overexpression being associated with inhibition of proliferation, migration and invasion (Wang L. et al., 2021). Collectively, it has the potential to act as a biomarker for GB. However, this association has only been reported by (Wang L. et al., 2021) and requires further research to validate findings. Other studies have suggested this miRNA as a biomarker for the diagnosis of other types of cancer, including osteosarcoma and lung adenocarcinoma (Huang et al., 2019; Li Y. et al., 2024), providing a basis for its utility as a biomarker. In addition, miR-6784-5p, miR-4276 and miR-4648, which were ranked in the top 100 by each of the five feature selection methods in this study, have not previously been reported as biomarkers for glioma, therefore, they represent potentially novel biomarkers that require further research to validate their clinical relevance. (Chen and Dhahbi, 2022) used ML to determine a panel of five miRNAs that could differentiate between cancer and non-cancer samples. miR-663a and miR-6784-5p were two of the miRNAs identified in this panel. Machine learning approaches also predicted miR-6784-5p to be a biomarker in ovarian and gastric cancer (Hamidi et al., 2023; Wang L. et al., 2021; Huang et al., 2019; Li Y. et al., 2024; Chen and Dhahbi, 2022; Wang et al., 2025). miR-4276 was identified as a circulating diagnostic biomarker of gastric cancer using bioinformatics and validated in human blood samples (Li et al., 2025; Yoshikawa et al., 2023) determined that the upregulation of miR-4648 was a biomarker for the recurrence of colorectal cancer, but to the best of our knowledge, there are no reports on its role in glioma. In addition, a machine learning model identified miR-4648 as a diagnostic biomarker of pancreatic cancer (Chi et al., 2023). Although these miRNAs have not been associated with glioma before, the results from this study and the fact that they have been shown to be biomarkers for other cancers provide a basis for future research into their clinical validity as glioma biomarkers. It is important to distinguish between diagnostic biomarkers and mechanistic drivers. Diagnostic biomarkers are used primarily to indicate the presence of disease and support detection and patient stratification based on consistent statistical associations. In contrast, mechanistic drivers actively contribute to disease biology and may represent therapeutic targets; however, establishing such roles requires functional validation beyond correlative analyses. Therefore, this study focuses on the diagnostic value of circulating miRNAs for glioma identification, while acknowledging that leading candidates warrant further mechanistic investigation.

From a clinical translation perspective, the identified miRNA panels may be amenable to established RT-qPCR and droplet digital PCR (ddPCR) workflows. This would typically involve standardized steps for biofluid collection and handling, RNA extraction, reverse transcription, and amplification using miRNA-specific primers (e.g., LNA-based assays) with suitable endogenous or exogenous controls for normalization. Prior work has demonstrated that miRNA signatures discovered using high-throughput profiling can be transitioned to targeted qPCR/ddPCR validation with acceptable analytical reproducibility for panels of modest size, supporting feasibility for future assay development and independent clinical validation (Mustafov et al., 2025; Yu et al., 2025).

To provide additional biological context for the potentially novel biomarkers (miR-663a, miR-6784-5p, miR-4276, and miR-4648), we explored predicted targets using miRDB and investigated enriched pathways using miRPath v4.0 (KEGG). Table 8 lists the top 10 predicted gene targets of each miRNA from miRDB. Pathway enrichment suggested that miR-663a, miR-4276 and miR-4648 are involved in axon guidance and nervous system development, while miR-4648 was additionally predicted to be involved in brain development. These predictions motivate follow-up validation (e.g., cross-checking targets in TargetScan and verifying downstream pathway perturbations in glioma). In addition to KEGG pathway context, functional enrichment analysis highlighted biological processes linked to transcriptional regulation, nervous system development, phosphorylation-related signalling, and axon guidance (Tables 9–12). Notably, the enrichment of axon guidance and nervous system development is consistent with the neural origin of glioma. Across all four GEO datasets, miR-6784-5p, miR-4276 and miR-4648 showed consistent downregulation in glioma, while miR-663a was consistently upregulated (Table 13); all comparisons were statistically significant after multiple testing correction (adjusted $p\leq 4.71\times 10^{-4}$ in all datasets). Similar analysis of the top 20 miRNAs of each of the five feature ranking algorithms resulted in 2 common miRNAs, miR-633a and miR-125a-3p. In the case of multiclass classification (glioma vs. meningioma vs. controls) none of the common miRNAs (miR-125a-3p, miR-4648, miR-4763-3p, miR-6786-3p, miR-6799-5p, miR-92b-5p) from the top 100 of each feature ranking algorithm are known clinically relevant biomarkers for meningioma or have any known association with meningioma.

**TABLE 8:** Binary classification on validation data (GSE211692) without SMOTE.

ML method	Best FS	Class	ACC	F1-score	Precision	Recall	AUC
LR	RF (10)	Glioma	99.32	92.22	86.81	98.34	99.98
​	​	Controls	99.32	99.64	99.93	99.36	99.98
​	​	All	99.32	92.22	86.81	98.34	99.98
KNN	ReliefF (10)	Glioma	99.22	91.25	84.21	99.59	99.78
​	​	Controls	99.22	99.59	99.98	99.20	99.78
​	​	All	99.22	91.25	84.21	99.59	99.78
DT	RFE (10)	Glioma	97.11	73.93	58.64	100.00	99.68
​	​	Controls	97.11	98.47	100.00	96.99	99.68
​	​	All	97.11	73.93	58.64	100.00	99.68
RF	ReliefF (10)	Glioma	99.44	93.54	88.52	99.17	99.99
​	​	Controls	99.44	99.71	99.96	99.45	99.99
​	​	All	99.44	93.54	88.52	99.17	99.99
SVM	mRMR (10)	Glioma	98.98	88.93	80.07	100.00	99.99
​	​	Controls	98.98	99.47	100.00	98.94	99.99
​	​	All	98.98	88.93	80.07	100.00	99.99
XGB	RF (10)	Glioma	99.35	92.66	86.64	99.59	99.98
​	​	Controls	99.35	99.66	99.98	99.34	99.98
​	​	All	99.35	92.66	86.64	99.59	99.98
LR	7 miRNA signatures	Glioma	99.68	93.02	99.59	96.19	99.76
​	​	Controls	99.68	99.98	99.68	99.83	99.76
​	​	All	99.68	96.50	99.63	98.01	99.76

**TABLE 9:** Binary classification on validation data (GSE211692) with SMOTE.

ML method	Best FS	Class	ACC	F1-score	Precision	Recall	AUC
LR	RFE (10)	Glioma	99.35	92.58	87.45	98.34	99.98
​	​	Controls	99.35	99.66	99.93	99.39	99.98
​	​	All	99.35	92.58	87.45	98.34	99.98
KNN	RFE (10)	Glioma	99.25	91.54	85.30	98.76	99.77
​	​	Controls	99.25	99.61	99.98	99.20	99.77
​	​	All	99.25	91.54	85.30	99.22	99.77
DT	LASSO (10)	Glioma	97.52	76.60	62.40	99.17	99.40
​	​	Controls	97.52	98.69	100.00	96.89	99.40
​	​	All	97.52	76.60	62.40	99.17	99.40
RF	LASSO (10)	Glioma	99.51	94.28	89.85	99.17	99.99
​	​	Controls	99.51	99.74	99.96	99.53	99.99
​	​	All	99.51	94.28	89.85	99.17	99.99
SVM	RF (10)	Glioma	99.17	90.77	83.10	100.00	99.99
​	​	Controls	99.17	99.56	100.00	98.94	99.99
​	​	All	99.17	90.77	83.10	100.00	99.99
XGB	RFE (10)	Glioma	99.51	94.30	89.55	99.59	99.99
​	​	Controls	99.51	99.74	99.98	99.50	99.99
​	​	All	99.51	94.30	89.55	99.59	99.99
LR	7 miRNA signatures	Glioma	99.68	93.02	99.59	96.19	99.76
​	​	Controls	99.68	99.98	99.68	99.83	99.76
​	​	All	99.68	96.50	99.63	98.01	99.76

**TABLE 10:** Top 10 predicted gene targets for candidate novel miRNA biomarkers (miRDB).

miRNA	Top predicted targets
miR-663a	ABO, ESPN, SCRT1, MIA2, ACSL3, NFIX, TGFB1, TNFRSF8, SLC29A3, AP5Z1
miR-4648	LOC403312, CLEC12A, PAXBP1, TMEM155, TNPO3, CD164, TSPOAP1, TRPM3, C10orf53, TMEM216
miR-6784-5p	LBX1, FOXE3, IMPAD1, IGFALS, SCRT2, SAXO1, FAM19A5, RBFA, MFAP3L, HAGH
miR-4276	Rictor, CDKN1B, RPS6KA3, AKT3, CCDC169-SOHLH2, ITGB6, BIRC6, LZTR1, SORL1, TNKS2

**TABLE 11:** Top enriched pathways/processes for miR-663a.

Pathway/Process	Merged p -value	Merged FDR
Negative regulation of transcription by RNA polymerase II	$7.23\times 10^{-13}$	$7.09\times 10^{-12}$
Positive regulation of transcription by RNA polymerase II	$8.34\times 10^{-13}$	$7.09\times 10^{-12}$
Regulation of transcription by RNA polymerase II	$7.45\times 10^{-11}$	$4.22\times 10^{-10}$
Regulation of transcription, DNA-templated	$5.26\times 10^{-9}$	$2.23\times 10^{-8}$
Nervous system development	$1.50\times 10^{-8}$	$5.12\times 10^{-8}$
Protein phosphorylation	$1.68\times 10^{-7}$	$4.16\times 10^{-7}$
Phosphorylation	$1.94\times 10^{-7}$	$4.16\times 10^{-7}$
Positive regulation of transcription, DNA-templated	$1.96\times 10^{-7}$	$4.16\times 10^{-7}$
Multicellular organism development	$4.17\times 10^{-6}$	$7.87\times 10^{-6}$
Negative regulation of transcription, DNA-templated	$4.94\times 10^{-6}$	$8.39\times 10^{-6}$
Actin cytoskeleton organization	$6.10\times 10^{-6}$	$9.42\times 10^{-6}$
Regulation of AMPA receptor activity	$1.14\times 10^{-5}$	$1.61\times 10^{-5}$
Protein autophosphorylation	$1.77\times 10^{-5}$	$2.32\times 10^{-5}$
Platelet activation	$2.23\times 10^{-5}$	$2.71\times 10^{-5}$
Anterior/posterior pattern specification	$3.70\times 10^{-5}$	$4.20\times 10^{-5}$
Axon guidance	$4.14\times 10^{-5}$	$4.40\times 10^{-5}$
Negative regulation of transforming growth factor beta receptor signaling pathway	$5.21\times 10^{-5}$	$5.21\times 10^{-5}$

**TABLE 12:** Top enriched pathways/processes for miR-4648.

Pathway/Process	Merged p -value	Merged FDR
Axon guidance	$2.54\times 10^{-11}$	$3.81\times 10^{-10}$
Flavonoid glucuronidation	$4.16\times 10^{-8}$	$3.12\times 10^{-7}$
Multicellular organism development	$2.16\times 10^{-7}$	$1.08\times 10^{-6}$
Nervous system development	$6.91\times 10^{-7}$	$2.59\times 10^{-6}$
Negative regulation of transcription by RNA polymerase II	$2.58\times 10^{-6}$	$4.98\times 10^{-6}$
Learning	$2.07\times 10^{-6}$	$4.98\times 10^{-6}$
Positive regulation of transcription by RNA polymerase II	$2.66\times 10^{-6}$	$4.98\times 10^{-6}$
Xenobiotic glucuronidation	$1.71\times 10^{-6}$	$4.98\times 10^{-6}$
Vesicle docking	$1.10\times 10^{-5}$	$1.83\times 10^{-5}$
Synaptic vesicle fusion to presynaptic active zone membrane	$1.66\times 10^{-5}$	$2.49\times 10^{-5}$
Phosphorylation	$1.89\times 10^{-5}$	$2.57\times 10^{-5}$
Regulation of transcription involved in G1/S transition of mitotic cell cycle	$2.63\times 10^{-5}$	$3.29\times 10^{-5}$
Angiogenesis involved in wound healing	$2.95\times 10^{-5}$	$3.40\times 10^{-5}$
Brain development	$4.53\times 10^{-5}$	$4.85\times 10^{-5}$
Cell adhesion	$4.86\times 10^{-5}$	$4.86\times 10^{-5}$

**TABLE 13:** Top enriched pathways/processes for miR-6784-5p.

Pathway/Process	Merged p -value	Merged FDR
Positive regulation of transcription by RNA polymerase II	$2.95\times 10^{-10}$	$1.77\times 10^{-9}$
Regulation of transcription, DNA-templated	$7.52\times 10^{-9}$	$2.26\times 10^{-8}$
Negative regulation of transcription by RNA polymerase II	$1.49\times 10^{-8}$	$2.99\times 10^{-8}$
Regulation of transcription by RNA polymerase II	$6.13\times 10^{-7}$	$8.35\times 10^{-7}$
Positive regulation of transcription, DNA-templated	$6.96\times 10^{-7}$	$8.35\times 10^{-7}$
Multicellular organism development	$9.57\times 10^{-7}$	$9.57\times 10^{-7}$

## 6 Limitations

A limitation of this study concerns the metadata for dataset GSE113740. Review of the available annotations confirms that the 25 tumour samples are labelled only as “disease status: glioma,” with both pathological grade and pathological T stage listed as “uncertain.” This precludes reliable subtype assignment (e.g., glioblastoma versus IDH-mutant and 1p/19q-codeleted oligodendroglioma). Accordingly, these samples were analysed as a single glioma group.

We acknowledge that this introduces biological heterogeneity, as glioma subtypes differ substantially in molecular features, clinical behaviour, and prognosis. Pooling samples may therefore dilute subtype-specific miRNA signals, particularly between highly aggressive entities such as glioblastoma and more favourable tumours such as 1p/19q-codeleted oligodendrogliomas. Despite this limitation, our miRNA panels consistently discriminated glioma from controlsacross datasets (AUC >0.95 ), supporting their utility for broad glioma detection. Future work will prioritise molecularly stratified cohorts aligned with the WHO 2021 integrated histo-molecular classification (e.g., TCGA-GBMLGG) to enable refined subtype-level analyses.

In addition, age, sex, inflammation, or comorbidities need to further be investigated as other key factors that may affect circulating miRNA profiles. In specific, age-related differences in plasma miRNA expression have been previously reported, with brain-enriched miRNAs such as miR-134 showing peak levels in mid-life, with sex-specific patterns (Sheinerman et al., 2018; La Grotta et al., 2025). Sex-related dimorphism has also been observed, including female-specific downregulation of miR-4297 in glioma (Xu et al., 2022). In addition, systemic inflammation can increase circulating levels of miRNAs such as miR-21, miR-155, and miR-122, while comorbidities associated with physiological stress or organ dysfunction (e.g., frailty or renal impairment) have been linked to elevated miR-23a-5p and miR-26a-5p (Vibo et al., 2024; Xu et al., 2022). Due to the lack of relevant meta-data information on the evaluated datasets the above factors have not been considered in the present study.

Finally, the multi-class classification included a limited number of meningioma samples (n = 17). Although SMOTE was employed to address this class imbalance, synthetic oversampling cannot fully replicate the true biological diversity and complexity of real clinical samples. This limitation may also affect the stability and biological interpretability of meningioma-related feature rankings. Therefore, the performance metrics for this specific class should be interpreted cautiously and further validated using larger cohorts. Moreover, independent validation of the multiclass classification setup with meningioma class was not feasible because the independent dataset GSE211692 does not have meningioma samples, and thus the generalizability of the selected miRNA signatures when meningioma samples are included could not be validated.

## 7 Conclusion

This study identified a panel of key miRNA signatures that can classify glioma subtypes and assessed their predictive accuracy using multiple machine learning models. Leveraging GEO blood serum miRNA expression data, the proposed pipeline integrated pre-processing, feature selection, ML classifiers, and SMOTE-based imbalance handling. The selected miRNAs enabled near-perfect binary classification (F1-score, AUC = 1.00) and robust multi-class performance (F1-scores 0.97–0.99, AUC 0.99), with KNN consistently achieving the highest scores. Several miRNAs identified in this study, including miR-328, miR-4763-3p, miR-873, miR-769-3p, miR-125a-3p, miR-1914-5p, and miR-1225-3p, have been previously reported as glioma biomarkers, supporting the clinical relevance of our findings. Importantly, miR-663a, miR-6784-5p, miR-4276, and miR-4648 ranked as top 100 by each of five feature selection techniques are potential novel biomarkers for glioma, with no prior reports linking them to the disease. While these miRNAs have been implicated in other cancer types, their identification here provides a foundation for future glioma-specific biomarker validation. Our findings demonstrate that targeted miRNA selection combined with optimised predictive modelling can achieve high diagnostic accuracy with compact feature sets, supporting feasibility for clinical deployment. Limitations include the small meningioma sample size and possible batch effects, which warrant validation in larger, diverse cohorts. Future work should explore multi-omics integration, deep learning–based models, and clinical translation into standardised, non-invasive diagnostic protocols.

## Data Availability

The data used in this study are from publicly available datasets described in the Subsection 3.1.
